# Multiple GPCR Functional Assays Based on Resonance Energy Transfer Sensors

**DOI:** 10.3389/fcell.2021.611443

**Published:** 2021-05-10

**Authors:** Yiwei Zhou, Jiyong Meng, Chanjuan Xu, Jianfeng Liu

**Affiliations:** ^1^Cellular Signaling Laboratory, Key Laboratory of Molecular Biophysics of Ministry of Education, College of Life Science and Technology, Huazhong University of Science and Technology, Wuhan, China; ^2^Bioland Laboratory, Guangzhou Regenerative Medicine and Health Guangdong Laboratory, Guangzhou, China

**Keywords:** GPCR, functional assay, G-protein, β-arrestin, dimerization, BRET, TR-FRET

## Abstract

G protein-coupled receptors (GPCRs) represent one of the largest membrane protein families that participate in various physiological and pathological activities. Accumulating structural evidences have revealed how GPCR activation induces conformational changes to accommodate the downstream G protein or β-arrestin. Multiple GPCR functional assays have been developed based on Förster resonance energy transfer (FRET) and bioluminescence resonance energy transfer (BRET) sensors to monitor the conformational changes in GPCRs, GPCR/G proteins, or GPCR/β-arrestin, especially over the past two decades. Here, we will summarize how these sensors have been optimized to increase the sensitivity and compatibility for application in different GPCR classes using various labeling strategies, meanwhile provide multiple solutions in functional assays for high-throughput drug screening.

## Introduction

G protein-coupled receptors (GPCRs) represent one of the largest membrane receptor superfamily, which is encoded by approximately 3% of human genes and over 800 members (Klabunde and Hessler, 2002; Fredriksson et al., 2003; Pin et al., 2019). They are widely expressed in all cells and organs from brain tissue to blood vessels, and are responsible for sensing a variety of external stimuli, ranging from light and temperature to neurotransmitters, peptides, and lipids (Lagerstrom and Schioth, 2008). GPCRs are involved in diverse physiological activities and play critical roles in pathogenesis, making them important drug targets (Sriram and Insel, 2018).

Members of the GPCR superfamily share a common seven-transmembrane (7TM) topology, and are classified into classes A, B, C, and F according to sequence similarity (Fredriksson et al., 2003; Fredriksson and Schioth, 2005). Generally, class A GPCRs possess a short extracellular domain (ECD), while class C GPCRs have a large ECD called Venus Flytrap domain (VFT). Ligand binding induces conformational changes from the extracellular ligand-binding site to the intracellular side of the receptor. The outward movement of the cytoplasmic end of transmembrane domain (TM) 6 in class A GPCRs opens up an intracellular cavity to accommodate the Gα subunit and activate G protein; in class B GPCRs, TM6 shows a disruption of the helical fold and the formation of a sharp kink to bind Gα subunits (Hilger et al., 2020). In contrast to Class A and B GPCRs, which are reported to function as monomers, class C GPCRs are reported as obligatory dimers (Kniazeff et al., 2011). Ligand binding to class C GPCR leads to the closure of VFT, triggering the conformational change in the cysteine-rich domain or stalk domain, further rearranging the TMs from inactive interface to TM6/TM6 active interface, which is similar in class C GPCR homodimers such as metabotropic glutamate receptor type 2 (mGlu2) (Xue et al., 2015), mGlu5 (Koehl et al., 2019), and calcium sensing receptor (CaSR) (Liu et al., 2020), or heterodimer, like metabotropic γ-aminobutyric acid receptors (GABA_*B*_ receptor) (Xue et al., 2019; Mao et al., 2020; Papasergi-Scott et al., 2020; Park et al., 2020; Shaye et al., 2020). Hence, monitoring the conformational changes of GPCRs provide a structural basis to evaluate GPCR activation.

**FIGURE 1:**
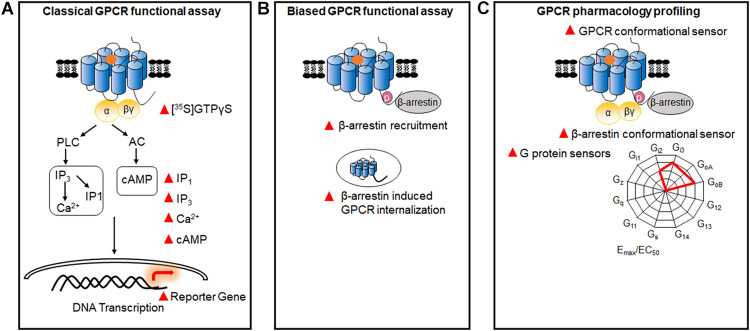
GPCR functional assays. **(A)** Classical GPCR functional assays based on the measurement of GTPγS, and Ca^2+^, IP1, IP3, cAMP, and reporter gene. **(B)** Biased GPCR functional assays. Assays were used to identify the biased signals between G proteins and β-arrestins, through detecting β-arrestin recruitment and GPCR internalization. **(C)** Multiple GPCR pharmacology profiling. GPCR sensors based on the conformational changes of different G protein subtypes, β-arrestins, and GPCRs.

The classical functional assays used to measure the activity of GPCRs are mainly based on downstream messengers, such as Ca^2+^ release, 1, 4, 5-inositol phosphate (IP3)/IP1 accumulation, cyclic adenosine monophosphate (cAMP) production, or reporter gene expression (Thomsen et al., 2005). Most of them have been successfully developed into high-throughput screening (HTS) and robust assays, and widely applied in the pharmaceutical industry and academic research (Figure 1A). Furthermore, with the discovery of G protein-independent β-arrestin signaling, functional assays have been developed by detecting β-arrestin recruitment to GPCRs or β-arrestin-induced GPCR internalization (Zhang and Xie, 2012; Figure 1B), while the first GPCR biased drug have been approved by FAD recently (Mullard, 2020). In addition to these classical assays, multiple functional assays based on resonance energy transfer (RET), which is a technology to detect the protein-protein interaction, have been developed in recent years for directly monitoring conformational changes in GPCRs, G proteins, and β-arrestins (Figure 1C). Using these sensors, the GPCR signaling profiles and GPCR activation process have been investigated at multiple scales.

RET sensors have been well-reviewed to illustrate GPCR activation and signaling previously (Lohse et al., 2012; Kauk and Hoffmann, 2018; El et al., 2019; Haider et al., 2019; Quast and Margeat, 2019). In this review, we have summarized the FRET and BRET sensors, which contributed to G protein and β-arrestin signaling, intra-GPCR rearrangement, and inter-GPCR movement investigations, especially in recent years. Meanwhile, we will focus on how these sensors are optimized to better investigate GPCR signaling and adapted to HTS in functional assays, as well as what new mechanism have been identified based on these sensors.

## The Principles of RET

**FIGURE 2:**
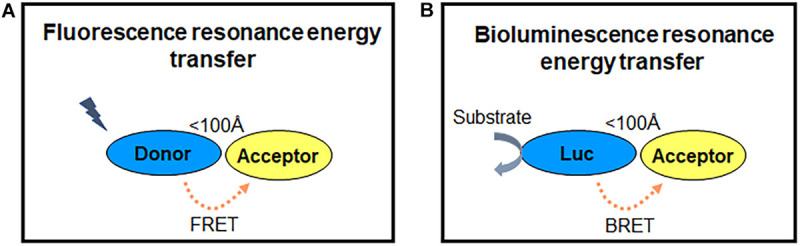
RET principle. **(A,B)** FRET and BRET detect energy transfer between two proteins within 100 Å, while one fluorophore is excited by laser as energy donor in FRET **(A)** and enzymes (luciferase) reacting with substrate (coelenterazine) to emit light as the energy donor in BRET **(B)**. The energy acceptor is another fluorophore in both FRET and BRET.

RET is a photo physical process, in which the energy of a fluorescent donor is transferred to a suitable fluorescent energy acceptor (Förster, 1948; Figure 2). The efficiency of RET depends on three parameters: (1) the emission spectrum of the donor overlaps with the excitation spectrum of the acceptor; (2) the distance between the fluorophores is within 100 Å; (3) the relative orientation of their dipole moments toward each other (the parallel dipole orientation gets highest RET) (Stryer, 1978). According to the fluorescent labels, RET sensors can be normally classified into FRET and BRET. The excitation of FRET donor fluorophores needs an extra excitation laser, while BRET is based on the use of light-emitting enzymes-luciferase and other different variations. RET is a good approach to measure the GPCR activation and signaling in a living system (Cottet et al., 2012; Lohse et al., 2012; Kauk and Hoffmann, 2018).

## Gpcr/G Protein Signaling Functional Assays

### Sensors for GPCR/G Protein Interaction

**FIGURE 3:**
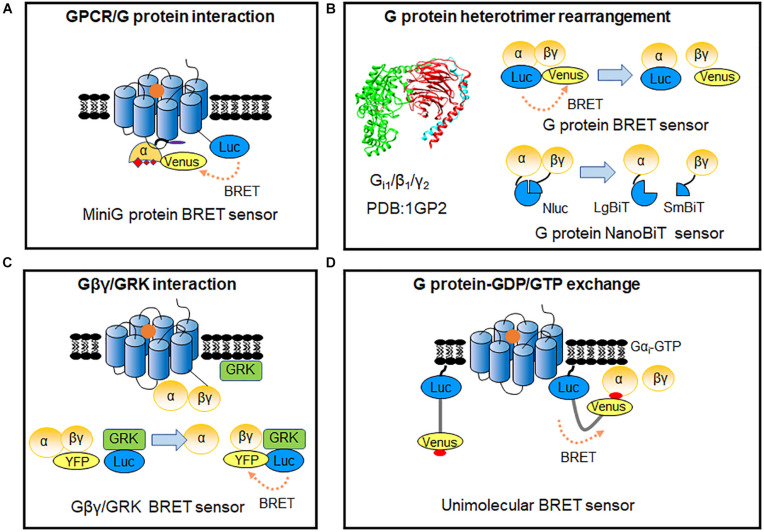
RET-sensors for GPCR/G protein interaction. **(A)** MiniG protein BRET sensor. MiniG protein was introduced into BRET sensors for G protein recruitment detection. **(B)** G protein heterotrimer-based sensor. Luciferase and YFP-tagged Gα-Gβγ constitute the G protein intermolecular BRET sensor for detecting the rearrangements of G protein heterotrimers (upper). G protein heterotrimer NanoBiT sensor based on Nluc complementation system was applied to monitor G protein heterotrimer rearrangements (lower). **(C)** Gβγ-GRK BRET sensor. The BRET signal was produced between the Gβγ and GRK after GPCR activation. **(D)** Unimolecular BRET sensor, BERKY biosensor (BRET biosensor with ER/K linker and YFP). After G protein activation, the detector module YFP-KB-1753 binds to active G-protein Gα_*i*_-GTP on membranes, and the BRET signal produced between N-terminus Nluc and YFP.

G protein heterotrimer activity is initiated by exchange of GDP with GTP, when ligand binding to GPCR triggers the G protein coupling to the receptor (Neubig, 1994). Though some RET-based sensors have used to detect the dynamic interaction of G proteins to GPCRs upon stimulation (Table 1; Azpiazu and Gautam, 2004; Gales et al., 2005; Hein et al., 2005; Nobles et al., 2005; Philip et al., 2007; Audet et al., 2008), agonist-induced G protein recruitment represents high diversity and specificity among different G protein subtypes and GPCRs (Du et al., 2019) and dynamic GPCRs and G proteins interactions cannot always easily be detected. MiniG proteins are used to improve the stability of the GPCR-G protein complex (Nehme et al., 2017). They are modified by deleting membrane anchor domains and the Gβγ binding surface in wild-type Gα proteins, and mutated in many positions to increase GPCR/G protein complex stabilization (Nehme et al., 2017; Wan et al., 2018). MiniG BRET sensors used energy pair of *Renilla* luciferase (Rluc) and Venus fused in GPCR and miniG protein, respectively (Figure 3A), which can effectively recognize different families of GPCRs, even class F GPCRs (Wright et al., 2019). MiniG BRET sensors can be used to investigate the dynamic interactions of GPCRs with almost all G protein families, including Gα_*i/o*_, Gαs, Gα_12/13_, and Gα_*q/11*_ (Wan et al., 2018). And then, the miniG_*q*_ sensor is successfully applied in HTS compare of 5-HT_2A_ serotonin receptor hallucinogen agonists (Kim et al., 2020). Further optimization of miniG BRET sensors by replacing the BRET donor Rluc with NanoLuc (Nluc), which has stronger brightness and smaller size (Hall et al., 2012), or replacing the BRET donor and acceptor with NanoLuc Binary Technology (NanoBiT) pairs, which is based on the Nluc complementation system consisting of LgBiT (18 kDa) and SmBiT (1.3 kDa) proteins (Dixon et al., 2016), can further increase the sensitivity. The optimized miniG sensors can be used to detect GPCR activation in intracellular compartments, such as the Golgi apparatus (Wan et al., 2018).

**TABLE 1:** The typical sensors for GPCR and modulators.

Targetedmodulators	Donor-acceptor pairs	Comments	Receptor/modulator	References
**Gprotein**				
GPCR-G protein	CFP-YFP	The FRET sensor illustrated M2R and G_o_ protein form the exclusive complex after agonist stimulation at membrane.	M2R/Gα_o_-Gβ.	Azpiazu and Gautam, 2004
	Rluc-GFP	BRET sensor measuredthe direct and kinetic interaction of Gα_s_β_1_γ_2_ proteins and β_2_AR in living cells, and supported the precoupling of Gα_s_β_1_γ_2_-β_2_AR by basal BRET signal.	β_2_AR/Gα_s_β_1_γ_2_	Gales et al., 2005
	CFP-YFP	The FRET sensor detected the fast agonist-induced α_2A_AR- Gα_i1_β_1_γ_2_ interactionkinetic in single living cell (<100 ms).	α_2A_AR/Gα_i1_β_1_γ_2_	Hein et al., 2005
	CFP-YFP	The FRET sensor identified α_2A_AR pre-coupled to G_o_ protein but not G_s_,while IP pre-coupled to G proteins in opposite way, indicating the specific interaction between GPCRs and G proteins.	α_2A_AR/Gα_o_/Gα_s_ IP/Gα_o_/Gα_s_	Nobles et al., 2005
	eCFP-eGFP	The FRET sensorobserved B_2_R pre-coupled to Gα_q_βγprotein in the resting statewhich allowed for a rapid and directed cell response.	B_2_R/Gα_q_	Philip et al., 2007
	Rluc8-Venus/Nluc-Venus	The miniG BRET sensor is modified from native G protein,obtained higher stability and selectivity. The miniG BRET sensors can recognize and stabilize the active states of β_2_AR and several Frizzled paralogs.	β_2_AR/miniGα_s_ β_2_AR/miniGα_12_ Frizzled receptors/miniGα	Wan et al., 2018; Wright et al., 2019
Gβγprotein-GRK	Nluc-Venus	These sensors can quantitatively detectthe magnitudes and kinetics of GPCRs general Gβγ-GRK interaction allowed the fingerprinting to be profiled of individual GPCR.	Gβγ-masGRK3ct	Masuho et al., 2015
Gprotein-specific unimolecules	Nluc-YFP	Membrane-anchored unimolecular BRET sensor specifically binds to GTP-Gαprotein and produce the BRET signal to indicate the activity of endogenous GPCRs/G proteins without any modifications.	α_2A_AR/Gα_i_ M3R/Gα_q_ PAR1/Gα_13_	Maziarz et al., 2020
Gαβγ heterotrimer rearrangement	CFP-YFP	The G protein heterotrimer FRET sensor measured α_2A_AR ligand-induced G protein activity, indicating the rearrangement occurred in Gα_i_β_1_γ_2_ heterotrimer instead of dissociation, and detecting activation of G protein in 1-2s, slower than receptors activation.	α_2A_AR/Gα_i_β_1_γ_2_	Bunemann et al., 2003
	Rluc-GFP10	The multiple sites inserted BRET sensorscan monitor conformational rearrangementsat Gα_i1_β_1_γ_2_subunits interfaces after α_2A_AR stimulation, supporting the open interface of Gα_i1_β_1_γ_2_ rather than totally dissociation.	α_2A_AR/Gα_i1_β_1_γ_2_	Galés et al., 2006
	Rluc8-GFP10	With high sensitivity, the BRET sensors re-defined the SII as the partial agonist of AT_1_R rather than β-arrestins biased agonist by detecting multiple G protein heterotrimersactivities.	AT_1_R/Gαβγ	Sauliere et al., 2012
	Rluc8-GFP	The study generated through exhaustive protein engineering and empirical testing, building the TRUPATH suite of Gαβγ biosensors includes the first Gα_15_ and Gα_*Gustducin*_ probes.	β_2_AR/Gαβγ κOR/Gαβγ CB_1_R/Gαβγ μOR/Gαβγ NT_1_R/Gαβγ	Olsen et al., 2020
	Nluc	The NanoBiT system is used for monitoring the most G protein heterotrimers kinetics in real-time with highly reproducible signals under most GPCRs, such as AT_1_R, D2R, Prostanoid receptors.	Prostanoid receptors/Gαβγ AT_1_R/Gαβγ β_2_AR/Gαβγ D2R/Gαβγ	Inoue et al., 2019
	CFP-YFP	FRET sensor of Gα_s_ protein heterotrimer exhibited the decrease in ratiometric FRET after 100 μM adenosine stimulation of A_2A_R, indicating α and βγ subunits of G_s_ dissociated or at least reoriented.	A_2A_R/Gα_s_β_1_γ_2_	Hein et al., 2006
	CFP-YFP	Using FRET-based assay developed the direct sensors in mammalian to measure multiple G protein subtypes heterotrimer changes, it is indicated that Gα_i_ and Gα_*z*_ undergo rearrangement rather than dissociation, whereas Gα_o_ dissociate or rearrange in distinct manner after α_2A_AR activation.	α_2A_AR/Gα_o/i/z_β_1_γ_2_	Frank et al., 2005
	YFP-mTurquoise	The improved G_q_FRET sensor (with the best CFP variant) firstly allowed the detection of K_*on*_ of G_q_ and the FRET sensor indicated the dissociation of G_q_from G protein heterotrimer after stimulation of H1R.	H1R/Gα_q_β_1_γ_2_	Adjobo-Hermans et al., 2011
	Venus-mTurquoise2	The Gα_13_ FRET sensor can be used to detect heterotrimeric Gproteinsactivity in HeLa and primary HUVECs,and the sensor confirmed the dissociation of Gα_13_ from Gαβγ complex under LPA2 receptor and PARs stimulation.	LPA2 receptor/Gα_13_β_1_γ_2_ PARs/Gα_13_β_1_γ_2_	Mastop et al., 2018
**β-arrestins**				
GPCR/β-arrestins	Rluc-YFP	The BRET sensor detected the β-arrestin2 interaction with β_2_AR after agonist addition with high sensitivity.	β_2_AR/β-arrestin2	Angers et al., 2000
	Rluc-eYFP	These BRET sensors were used to identify the interaction of β-arrestins with TRHRs, and then revealed TRHR1 interacted equally β-arrestin1 and 2 while TRHR2 only interacted with β-arrestin2 that correlated with β-arrestins promoted internalization of TRHRs.	TRHRs/β-arrestins	Kroeger et al., 2001; Hanyaloglu et al., 2002
	Rluc-YFP	Using OTR/β-arrestin BRET sensor obtained the result that the BERT signal started at 10s and achieved the peak at 35s, indicated the delay and slow course of β-arrestin recruitment may be limited by receptors phosphorylation via GRK.	OTR/β-arrestin2	Hasbi et al., 2004
	RlucII-rGFP	Enhanced bystander(Eb) BRET sensor monitored compartmental trafficking of β-arrestins/AT_1_R complex and compartmentalβ-arrestin recruitment induced by AT_1_R agonist with high spatial-temporal resolution in living cells, providing the clear imaging BRET signal.	AT_1_R/β-arrestin2	Namkung et al., 2016
	CFP-YFP	The FRET sensor was used to detect the dynamic GPCR/β-arrestins interaction and provided the evidence oftime delay compared to the activation of PTHR.	PTHR/β-arrestin2	Vilardaga et al., 2002, 2003
	CFP-GFP/YFP	The study used FRET sensors to classify the P2Y_2_ receptor as a class A receptor when stimulated with ATP or as a class B receptor when stimulated with UTP, according to the interaction manners with β-arrestins.	P2Y_2_R/β-arrestins	Hoffmann et al., 2008
	FlAsH/ReAsH	Double site-specific and orthogonal labeled FRET sensor was proposed in PTHR/β-arrestin2interaction investigation with minimal disturbance of their function proved the method may be widely applied.	PTHR/β-arrestin2	Zurn et al., 2010
β-arrestins conformational changes	Rluc-YFP/GFP	The double-brilliance β-arrestin intramolecular BRET sensor firstly allowed the real-time monitoring of conformational changes of β-arrestin2 after both classA (β_2_AR) and class B (V2R) receptors activation in intact cells.	β_2_AR/V2R β-arrestin2	Charest and Bouvier, 2003; Charest et al., 2005
	Rluc-YFP	The intramolecular BRET sensor observed the different conformational changes of β-arrestin2 upon stimulations of biased ligands or unbiased ligands under AT_1_R, β_2_AR and PTH1R.	β_2_AR/β-arrestin2 AT_1_R/β-arrestin2 PTH1R/β-arrestin2	Shukla et al., 2008
	Nluc-CyOFP1	The optimized BRET sensorsdetected β-arrestin2 partial active state under AT_1_R stimulations with increased brightness and wider spectral separation, and the sensors can be applied with a wide panel of class A and B receptors even orphan receptors.	AT_1_R/β-arrestin2	Oishi et al., 2020
	CFP-FlAsH	The FlAsH-FRET sensorshave advantages of the small size and robust fluorescence signal. The multiple sites inserted FlAsH-FRET sensors detected different conformational changes in β-arrestin2 under β_2_AR and M_2_AChR after agonist stimulation, and supportedreceptor-specific patterns of conformational changes in β-arrestin2.	β_2_AR/β-arrestin2 M_2_AChR/β-arrestin2	Nuber et al., 2016
	Rluc-FlAsH	A series of intramolecular FlAsH-BRET sensors were designed todetect different conformational changes in β-arrestins under multiple GPCRs, such as PTH_1_R, β_2_AR and AT_1_R, supporting that specific ligand-receptors could invoke different conformational changes in β-arrestins and provide the insight into mechanism of GPCRs generating diverse functions.	PTH_1_R/β-arrestin2 β_2_AR/β-arrestin2 AT_1_R/β-arrestin2	Lee et al., 2016

*TRHR, Thyrotropin-releasing hormone receptors; OTR, Oxytocin receptor; V2R, V2 vasopressin receptor; PTH1R, Parathyroid hormone 1 receptor; α_2A_ AR, α2A adrenergic receptor; β_2_AR, β_2_-Adrenergic receptor; P2Y_2_R, purinergic receptor; A_2A_R, A_2A_ adenosine receptor; mTurquoise, Monomeric Turquoise; H1R, Histamine H1 receptor; PARs, protease activated receptors; IP, prostacyclin receptor; κOR, κ-opioid receptor; μOR, μ-opioid receptor; CB_1_R, cannabinoid-1 receptor; NT_1_R, Neurotensin-1 receptor; D2R, Dopamine D2 receptor; B_2_R, B2-Bradykinin receptor; M3R, M3 muscarinic acetylcholine receptor; M2R, M2 muscarinic acetylcholine receptor.*

### Sensors for G Protein Heterotrimer

As G protein can pre-associate to some GPCRs in the absence of ligand (Gales et al., 2005; Nobles et al., 2005; Philip et al., 2007; Audet et al., 2008), detecting G protein heterotrimer activity provides a more general way to evaluate GPCR activation. Measuring the proximity change between Gα and Gβγ subunits through BRET assay (Figure 3B), can reflect the G protein heterotrimer states and activation of GPCRs (Galés et al., 2006). Three flexible regions in Gα_*i*_ around amino acid numbers 91, 122, and 60 were reported to be accessible for BRET sensors insertion, according to Gα subunit structure. Interestingly, the BRET ratio between Gα_*i*__1_-91Rluc or Gα_*i*__1_-122Rluc and green fluorescent protein (GFP) 10-Gγ decreased significantly, whereas Gα_*i*__1_-60Rluc and GFP10-Gγ exhibited an increase of BRET ratio during α_2A_ AR activation (Galés et al., 2006). This indicates that small rearrangements have occurred in the Gαβγ heterotrimer, which can be detected using BRET assay. Rluc can be replaced by Rluc8 and other enhanced luciferases (Rluc II) to increased brightness and quantum yield in BRET (Loening et al., 2006; Sauliere et al., 2012). Based on optimized BRET sensors, activation of different G protein subtypes can be measured, such as Gα_*i*_, Gα_*o*_, Gα_*s*_, Gα_*q*_, Gα_12_, and Gα_13_. GPCR ligand such as S II, which was previous known as β-arrestin-biased agonist of angiotensin II receptor type 1A (AT_1_R) using classical functional assays, is considered as partial agonist, as it can induce ∼20% BRET ratio change in G_*i/o*_, G_*q*_, and G_13_ compared with the full agonist Ang II (Sauliere et al., 2012). It indicated the advantage and the necessity to combine BRET sensor in GPCR functional assays and proved the HTS application of BRET sensors in G protein signaling.

The G protein BRET sensors can be useful tools to evaluate the activity of individual G protein subtypes and many sensors have been reported as summarized in Table 1. However, not all the subtypes can be detected with good sensitivity. The new BRET Gαβγ biosensors TRUPATH have systematically optimized the insertion positions of the donor in Gα and the best combination of Gβ and Gγ subtypes. 14 optimized sensors have been developed, including the first Gα_15_ and Gα_*Gustducin*_ sensors (Olsen et al., 2020). TRUPATH biosensors extremely increase the sensitivity of G protein BRET functional assays, contributing to the development of a powerful platform to investigate most G proteins activation in an array of GPCRs agonists, antagonists, inverse agonists and biased ligands. Meanwhile, NanoBiT system has been used to modify the G protein sensors (Figure 3B). The advantages of the smaller size and strong signal can avoid possible steric hindrance induced by large proteins such as Rluc, GFP or Venus, and enable the stable detection in hours (Dixon et al., 2016; Inoue et al., 2019).

To further compare the activation efficacy between different G protein subtypes, Gβγ-GRK NanoBRET sensor has been developed based on the Gβγ subunit and lipid-modified reporter peptide GRK3ct (masGRK3ct) (Hollins et al., 2009; Masuho et al., 2015; Figure 3C). The NanoBRET strategy was achieved by fusing Nluc to the GRK3ct and Venus to Gβγ (Masuho et al., 2015). It can determine both the kinetics and extent of G protein activation to independently analyze the catalytic activities of GPCRs and their signaling efficacy toward various targeted Gα protein subtypes (Masuho et al., 2015; Hauser et al., 2018).

Up to now, tools that are suitable for primary cells or native tissues of endogenous GPCRs remain limited. Very recently, Maziarz et al. (2020) developed a type of BRET biosensor with ER/K linker and YFP, called BERKY biosensor to capture the GTP form of the Gα protein (Figure 3D). The membrane-anchoring sequence-fused Nluc was used as the BRET donor, and the YFP-fused synthetic peptide KB-1753 served as the acceptor (Johnston et al., 2006). This unimolecular biosensor can specifically and sensitively bind to Gα_*i*_-GTP, and causes an increased BRET signal. It allows endogenous Gα-GTP and free Gβγ to be detected in primary living cells, and record the activation of G proteins in native, physiological environments. BERKY biosensors have been developed for endogenous Gα_*q*_-GTP, Gα_13_-GTP, free Gβγ, and Rho-GTP in cells via a similar strategy (Maziarz et al., 2020).

## GPCR/β-Arrestin Signaling Functional Assays

β-arrestins are considered to be prominent mediators of GPCR internalization, facilitating GPCR desensitization and the negative regulating G proteins (Lefkowitz, 1998). β-arrestins also act as key modulators of GPCRs to initiate G protein-independent signaling pathways (Lefkowitz and Shenoy, 2005). Based on the interaction with β-arrestins, GPCRs can be classified into class A and B. Class A GPCRs interact with β-arrestins rapidly and transiently, whereas class B GPCRs stably associate with β-arrestins with a higher affinity (Oakley et al., 2000; Hasbi et al., 2004). β-arrestins can adopt different conformational changes while interacting with phosphorylated GPCRs (Shukla et al., 2008). The dynamic of β-arrestin conformational rearrangement can be longer than GPCR/β-arrestins interaction (Nuber et al., 2016). Accordingly, there are two main types of biosensors for studying the kinetics of GPCR/β-arrestin signal based on BRET and FRET: intermolecular sensors used for monitoring GPCR/β-arrestin dynamic interactions and intramolecular sensors used for measuring β-arrestin conformational rearrangement (Table 1; Bertrand et al., 2002; Charest and Bouvier, 2003; Krasel et al., 2005).

### Sensors for GPCR/β-Arrestin Interaction

**FIGURE 4:**
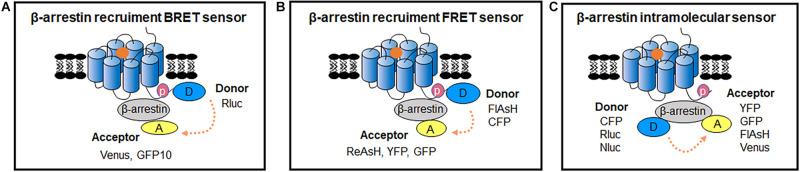
RET-sensors for GPCR/β-arrestin interaction and intramolecular β-arrestin conformational change. **(A)** Schematic representation of the BRET sensor for β-arrestin recruitment to GPCRs. Luciferase and YFP were introduced between β-arrestin and GPCRs, the BRET signal increased after GPCR activation. **(B)** β-arrestin recruitment FRET sensor. Different fluorophore pairs are introduced into FRET assay to better monitor β-arrestins dynamic recruitment to receptors in living cells. Donor-acceptor: FlAsH-ReAsH CFP-YFP CFP-GFP. **(C)** β-arrestin intramolecular sensor. The FRET-based and BRET-based intramolecular sensors are introduced in living cells to detect β-arrestins conformational changes, and multiple fluorophore pairs are applied. Donor-acceptor: CFP-FlAsH CFP-YFP CFP-GFP Rluc-YFP Rluc-GFP Rluc-Venus Nluc-Venus Nluc-YFP.

BRET sensors detecting recruitment of β-arrestins to active GPCRs in living cells was firstly reported by Angers et al. (2000). They used Rluc and GFP as BRET donor and acceptor, which were fused to the C terminus of β2 Adrenergic receptor (β_2_AR) and β-arrestin2, respectively (Figure 4A). The results showed a large increase in BRET ratio following β_2_AR stimulation, and represented an agonist concentration-dependent manner. Subsequently, many similar studies investigated the recruitment of β-arrestins to GPCRs (Table 1). Furthermore, this kind of BRET sensors were successfully applied in HTS for GPCRs antagonists, which show compatibility and sensitivity as a functional assay (Hamdan et al., 2005).

While the conventional BRET sensors use the non-natural combination of donor and acceptor from different species such as Rluc from *Renilla reniformis* and GFP from *Aequorea Victoria* to limit non-specific signals from random interaction, enhanced bystander BRET (EbBRET) sensor is composed with BRET energy pairs both from *Renilla reniformis*. It possesses moderate affinity, optimally transferred energy, and improved signal, which shows the advantage in investigation of compartmental and ligand-biased β-arrestin trafficking in the plasma membrane and endosomal membrane in real time (Molinari et al., 2008; Namkung et al., 2016). EbBRET sensors can work in real time image recording up to 20 min, greatly facilitating the studies of β-arrestin and GPCR trafficking at high spatial and temporal resolutions (Lan et al., 2012; Namkung et al., 2016; Cao et al., 2019).

Compared to BRET sensors, FRET sensors are more suitable for visualizing the kinetics of GPCRs and β-arrestins with high temporal resolution in cells (Lohse et al., 2012; Figure 4B). Using the FRET sensor, which consists of the CFP-labeled parathyroid hormone 1 receptor (PTH1R) and YFP-labeled β-arrestin2, has shown a time delay of β-arrestin2 recruitment to PTHR after receptor activation (Vilardaga et al., 2002, 2003). Through comparing the recruiting rate of β-arrestin2 to β_2_AR, repeated short-term stimulation promoted β_2_ARs phosphorylation and showed very rapid increase of FRET with t_0.__5_ at 2.1 s, while the first stimulation caused fairly slow FRET change (t_0.__5_ of 19.6 s), which indicate the GPCR phosphorylation is the rate-limiting step for β-arrestin recruitment (Krasel et al., 2005). Furthermore, β-arrestin subtypes: β-arrestin1 and β-arrestin2 can exist different recruitment manner to the same GPCR when stimulated with different ligand, such as P2Y_2_R (a Purine receptor subtype) agonist UTP induced both β-arrestin1 and β-arrestin2 interacted with P2Y_2_R, whereas ATP caused stronger interaction of β-arrestin1 than β-arrestin2 with P2Y_2_R (Hoffmann et al., 2008).

To reduce the size of FRET acceptor, a smaller fluorescent probe fluorescein arsenical hairpin binder (FlAsH) was introduced as FlAsH-FRET sensors (Figure 4B). A short peptide of 6 amino acids containing tetracysteine was inserted into the targeted protein, which can specifically bind to FlAsH dyes that produce FRET signal between proximate fluorophore pairs. The CFP/FlAsH FRET sensors showed almost five times the signal amplitude compared to the CFP/GFP FRET sensors (Hoffmann et al., 2005). And then, the double site-specific and orthogonal labeling FRET sensor such as FlAsH/ReAsH (a red arsenical hairpin binder) can also be introduced to investigate GPCR/β-arrestin dynamic interaction (Zurn et al., 2010).

### β-Arrestin Intramolecular Sensors

β-arrestin conformational change occurs following recruitment to the receptor (Charest et al., 2005; Nuber et al., 2016). β-arrestin intramolecular BRET biosensor is based on the proximity change between the N- and C-terminus of β-arrestin (Figure 4C). Intramolecular BRET sensors (Luc-β-arrestin-YFP) indicated that β-arrestin can adopt multiple active conformations with different ligands treatment (Shukla et al., 2008). It can be optimized by using Nluc and red-shifted fluorescent protein (CyOFP1) to increase brightness and wider spectral separation (Oishi et al., 2020). This sensor can monitor the early conformational changes of β-arrestin 2 in complex with GPCRs, with a wide panel of different class A and class B GPCRs upon agonist activation, and with orphan GPCRs known to spontaneously recruit β-arrestin2. After the R170E mutation was introduced, the sensor was able to detect the partial active state of β-arrestin2. It permits the monitoring of β-arrestin change in different stage during the GPCR activation (Oishi et al., 2020). Additionally, intramolecular FlAsH-BRET sensors using Rluc and FlAsH pair or intramolecular FlAsH-FRET sensors using CFP and FlAsH pair have also been developed (Figure 4C). They confirmed distinct conformational changes in β-arrestins induced by different ligands and GPCRs (Lee et al., 2016; Nuber et al., 2016; Strungs et al., 2019).

## Intramolecular Conformational GPCR Sensors

**FIGURE 5:**
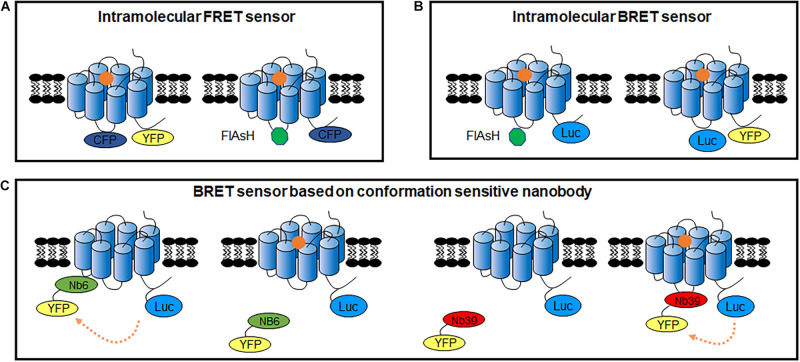
GPCR intracellular conformational changes sensors. **(A)** GPCR intracellular conformational changes sensors based on FRET between fluorescent protein (left) or FRET between CFP and FlAsH dye (right). **(B)** GPCR intracellular conformational changes sensors based on BRET between Rluc II and FlAsH dye (left) or BRET between Nluc and fluorescent protein (right). **(C)** GPCR intracellular conformational changes BRET sensors based on conformation sensitive nanobody. Nb6-YFP only binds to GPCR in inactivated state, while Nb39-YFP only binds to GPCR in activated state.

Though GPCR intramolecular RET sensors have already been developed in 1995 in purified GPCRs (Gether et al., 1995), Vilardaga et al. firstly reported a FRET sensor to detect GPCR conformational changes in living cells, which inserts CFP at the ICL3 and YFP at the C-terminus (Figure 5A) in PTHR and α_2A_ AR (Vilardaga et al., 2003, 2005). Using this sensor, the authors presented different FRET signals induced by full agonists (a strong decrease) and partial agonists (a weak decrease) or inverse agonists (a significant increase) of α_2A_ AR, indicating the dynamic activation process and distinct receptor conformation rearrangements specific to different ligands. Then, many similar intramolecular conformational GPCR sensors have been developed, such as β_1_AR (Rochais et al., 2007), β_2_AR (Reiner et al., 2010), A_2A_-adenosine receptor (A_2_AR) (Hoffmann et al., 2005), and B_2_-Bradykinin receptor (B_2_R) (Chachisvilis et al., 2006) as shown in Table 2. FlAsH labeling provided an alternative choice to replace CFP or YFP (Figure 5A). The FlAsH labeling (∼1 kDa) can be induced into the ICL3 of GPCRs as the energy acceptor, while inserting CFP as donor at the C-terminus. This FlAsH-FRET sensor confirmed the similar fast kinetics of GPCR activation and also showed a fivefold improvement in signal-to-noise ratio (Hoffmann et al., 2005).

**TABLE 2:** Multiple RET-sensors for detection of GPCR conformational changes.

Type of sensors	Fluorescent probe	Receptors	Comments	References
**RET-sensors applied in purified protein**				
Polarity sensitive fluorescent dyes	IANBD	β_2_AR	The first direct evidence for ligand specific conformational changes occurring in a GPCR.	Gether et al., 1995
Fluorescence quenching	Fluorescein maleimide (FM) and oxyl-N-hydroxysuccinimide as quencher	β_2_AR	Showed the relative movements of the two labeled amino acid and 20 s activation kinetics through the fluorescent change after ligand addition.	Ghanouni et al.,2001a,b
**Intramolecular RET-sensors in cells**				
FRET	CFP/YFP	PTHR; α_2A_ AR; β1AR; β_2_AR; A_2_AR; B_2_R	The first generation of FRET sensor to detect GPCR conformational changes in living cells, and showed a fast activation kinetic induced by various ligands in single cell assay using microscopy.	Vilardaga et al., 2003, 2005; Hoffmann et al., 2005; Chachisvilis et al., 2006; Rochais et al., 2007; Reiner et al., 2010
FRET	CFP/FlAsH	A_2_AR; α_2A_ AR	The smaller FlAsH tag avoided to disturb G protein signaling and provided a better signal-to-noise ratio compared with CFP/YFP FRET sensor.	Hoffmann et al., 2005
BRET	Rluc II/FlAsH	F prostanoid receptor; *AT1R;*β_2_AR	The BRET sensors allow microplate assay to probe the conformational rearrangement promoted by diverse ligands but require exogenous labeling and extensive washing.	Sleno et al., 2016; Bourque et al., 2017; Devost et al., 2017; Sleno et al., 2017
nanoBRET	Nluc/Halo-618	PTHR; α_2A_ AR; β_2_AR	These BRET sensors are directly translatable to other class A and B GPCRs and the high amplitude induced by agonist suitable for microtiter plate assays with HTS formats.	Schihada et al., 2018
nanoBRET	Nluc/YFP	β_2_AR	The genetically encoded biosensors don’t need dyes labeling and can be a tool to evaluate ligands with different intrinsic efficacy by BRET changes in HTS formats.	Picard et al., 2018
BRET	Rluc8/mVenus fused with conformation sensitive nanobody	KOR	Two KOR state specific nanobodies can be used as real time reporters for monitoring both ligand-dependent and independent conformational states in cells.	Che et al., 2020
nanoBRET	Nluc/NB80-GFP	β_2_AR	NanoBRET sensor based on NB80-GFP confirmed the activation of β_2_AR in VEGFR2-β_2_AR complexes was not influenced by VEGFR.	Kilpatrick et al., 2019
TR-FRET	SNAP (BG-green) /ACP17 (CoA–Lumi4-Tb)	LH receptor; GABA_*B*_R	Two cases illustrated how to use the TR-FRET toolbox construct intramolecular conformational change sensors.	Scholler et al., 2017a
**Intermolecular RET-sensors**				
FRET	CFP/YFP	mGlu1 homodimer	Observed the intermolecular FRET change between protomers and showed 10 ms of mGlu1 receptor activation kinetics between two subunits after agonist addition in real-time.	Tateyama and Kubo, 2006; Marcaggi et al., 2009
FRET	4-azido-L-phenylalanine (site specifically labeling Cy3 and Cy5 fluorophores)	mGlu2 homodimer	Used the unnatural amino acid-incorporation strategy to successfully constructed mGlu2 intermolecular sensor in living cells. And revealed new conformational states during receptor activation.	Liauw et al., 2021
BRET	Rluc/YFP	MT1/MT2 heterodimer	The specific interaction of ligands with the MT1/MT2 heterodimer was studied by this sensor.	Ayoub et al., 2004
TR-FRET	SNAP (BG -Green and BG-Lumi4-Tb)	mGlu2 homodimer	Inter-subunit FRET change induced by series ligands demonstrated that a reorientation of the ECDs is associated with receptor activation in mGlu receptor.	Doumazane et al., 2013; Scholler et al., 2017a

*IANBD, the cysteine-reactive fluorescent probe N,N^∗^-dimethyl-N-(iodoacetyl)- N^∗^-(7-nitrobenz2-oxa-1,3-diazol-4-yl) ethylenediamine; BG, O^6^-benzyl-guanines; metabotropic glutamate receptor (mGlu receptor); metabotropic γ-aminobutyric acid receptors (GABA_*B*_ receptor); luteinizing hormone (LH) receptor; β_2_AR, β_2_-Adrenergic receptor; B_2_R, B_2_-Bradykinin receptor; Parathyroid hormone 1 receptor (PTHR); α_2_A AR, α_2_A adrenergic receptor; A_2A_R, A_2A_-adenosine receptor; F prostanoid (FP) receptor; KOR, κ opioid receptor; D_2_R, D_2_-dopamine receptor; MT1, MT1 melatoninreceptor; MT2, MT2 melatonin receptor.*

Similar intramolecular BRET sensors of GPCRs were also used to monitor the conformational rearrangement promoted by diverse ligands (Sleno et al., 2016, 2017; Bourque et al., 2017; Devost et al., 2017). Intramolecular FlAsH-BRET sensors are modified the C-tail with Rluc II, and introduced FlAsH labeling in the ICL3 (Figure 5B). In the case of the F prostanoid receptor, AT1R, and β_2_AR, although three FlAsH-BRET sensors were constructed in a similar way, the BRET signals exhibited receptor-specific behavior, indicating that different GPCRs have unique conformational profile (Bourque et al., 2017). Furthermore, distinct patterns of conformational changes can be observed by biased ligand. Multiple positioned FlAsH-BRET sensors of AT_1_R showed rapid, sustained and robust BRET signals to allow the comparison of the full agonist and biased ligands stimulation (Devost et al., 2017). Moreover, intramolecular BRET sensors can be used to investigate the allosteric interactions between two receptors, such as the heterodimeric AT1R and prostaglandin F2α receptors (Sleno et al., 2017).

Alternatively, BRET assay with Nluc and YFP or GFP10 can be used to detect the GPCR conformational rearrangements (Figure 5B), which can be induced by various compounds or mutation in the receptor as well as the impact of interacting proteins (Picard et al., 2018). Schihada et al. (2018) screened the efficacy of different fluorescent proteins or dyes as acceptors, combined with Nluc as a BRET-based α_2A_ AR biosensor, NanoBRET 618 (Nluc and Halo Tag dye pair) showed the highest amplitude upon agonist stimulation. It is a powerful approach to distinguish slight differences induced by partial agonist in the BRET signal instead of the full agonist effect identified by classical cAMP assay. These BRET sensors are also adaptable for micro-liter plate assays with HTS formats.

A novel assay for detecting conformational changes in GPCRs, based on nanobodies recognizing specific conformations, has been reported in recent years. Several conformation-specific nanobodies for GPCRs have been developed, including κ opioid receptor (KOR) (Che et al., 2018), μ opioid receptor (Huang et al., 2015), M2-muscarinic receptors (Kruse et al., 2013), β_2_AR (Rasmussen et al., 2011), AT_1_R (Szalai et al., 2012), and mGlu2 (Scholler et al., 2017b). For example, two conformation-sensitive nanobodies of KOR, Nb39 and Nb6, recognize the active and inactive states, respectively (Che et al., 2018). Combined with the BRET approach, it can be used to detect KOR activity. For the Nb6 sensor, a strong BRET ratio decreased upon KOR activation induced by agonist, in which Nb6 dissociated from activated receptors and recovered after the antagonist addition, while the Nb39 sensor had the opposite effect (Figure 5C). Moreover, the conservative binding of Nb6 in the region provides a compatible tool for ligand-induced active conformational changes of other class A GPCRs, when replace their ICL3 by KOR ICL3 (Che et al., 2020). Nanobody-based GPCR conformational sensors also have the advantage of investigating the transactivation induced by other receptors. For example, Nb80, an active β2AR sensitive nanobody, was used to analyze the effect of vascular endothelial growth factor receptor 2 on β_2_AR activation (Kilpatrick et al., 2019). Nanobody application reduced the modification in GPCRs. However, as there are only a few nanobodies available for GPCRs and the intellectual property protection, the application of nanobodies in GPCR functional assays remains limited.

Regarding to the application in native cells or animals *in vivo* model, genetically encoded sensors based on GPCR conformational changes have incorporated circularly permuted fluorescent protein (cpGFP) to optical visualize the neurotransmitter release in brain (Sun et al., 2018; Peng et al., 2020). The cpGFP is modified from original GFP, in which the amino and carboxyl portions have been interchanged and reconnected by a short spacer between the original terminus. It is more flexible and accessible than original protein, and the fluorescence intensity of cpGFP is related to its conformation (Baird et al., 1999). Thus, cpGFP offered a suitable strategy for conformation-sensitive sensors. As similar movements between TM5 and TM6 occurred during class A GPCRs activation (Weis and Kobilka, 2018; Hilger et al., 2020), fusing cpGFP at the ICL3 would allow significant fluorescence signal changes of cpGFP following GPCR activation upon ligand binding (Doi and Yanagawa, 1999; Sun et al., 2018). Mutation to abolish the G protein coupling in these sensors is required to not change the physiological GPCR function when expressed in the animals. The advantage of genetically encoded sensors is the rapid and high resolution in two-photon imaging systems for spatial neurotransmitters detecting in living animal (Sun et al., 2018; Peng et al., 2020). However, to obtain sensors with high sensitivity, large number of screening has been done for the cpEGFP insertion and linker residues (Sun et al., 2018). The experience in developing the sensor of one neurotransmitter is not always well adapted to another. Neuromodulator sensors are available for dopamine, serotonin, norepinephrine, acetylcholine, endocannabinoid, adenosine and gastrin-releasing peptide, but for glutamate and γ-aminobutyric acid remains difficult (Labouesse and Patriarchi, 2021).

## Intermolecular Conformational GPCR Dimerization Sensors

Intermolecular FRET sensors are considered as good approaches for investigating the dimerization/oligomerization of GPCRs, especially in class C GPCRs (Milligan and Bouvier, 2005; Kniazeff et al., 2011). The classical CFP/YFP FRET sensor can detect the inter-subunit conformational change. When inserting the fluorescent protein in ICL2 of mGlu1 receptor, it showed an increased FRET signal indicating the relative movement of two mGlu1 subunits (Tateyama and Kubo, 2006). Subsequently, through measuring the FRET between two mGlu1 subunits in real-time; a fast increased inter-subunit FRET signal between protomers was detected within 10 ms after glutamate application (Marcaggi et al., 2009). However, difficulties remain in these classical CFP/YFP FRET sensor applications due to low sensitivity, photobleaching and limitations of inserting position.

Time-resolved FRET (TR-FRET) use long-fluorescence lifetime fluorophore, such as lanthanide cryptate instead of fluorescent protein. The fluorescence lifetimes of these molecules are very long, ranging from 100 to 1,000 μs, which leads the efficiency of FRET is not affected by the orientation of dipole moments between donor and acceptor, and becomes truly dependent on their distance (Mathis, 1995; Selvin, 2002). Taking a fixed delay time before acquiring the signal allows the removal of most of the fluorescent background provided by biological media and instrument, which largely improves the signal-to-noise ratio, compared with classical FRET sensors (Maurel et al., 2008; Scholler et al., 2017a). TR-FRET sensors can be adapted in multi-well plates format from 96 well to 384 well for drug HTS (Scholler et al., 2017a; Liu et al., 2020).

**FIGURE 6:**
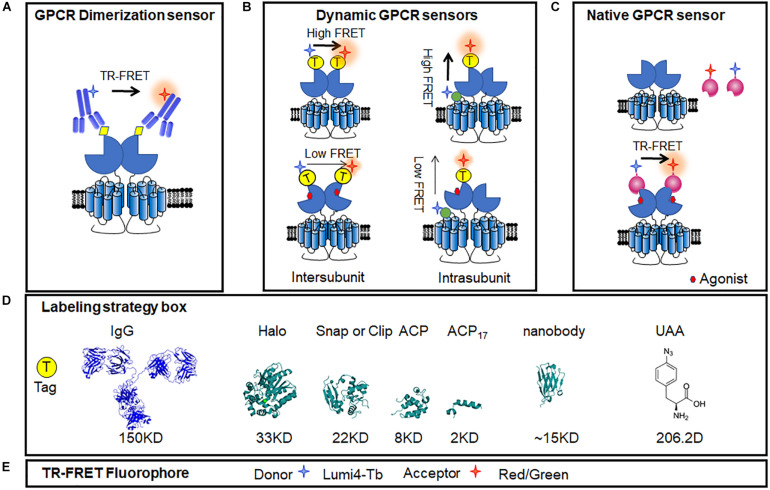
Intermolecular GPCR dimerization sensors. Fluorescent donors and acceptors were introduced using antibodies or tags allowing for the FRET measurement of GPCR dimerization and activation in membrane and living cells, even in the acutely isolated cells. **(A)** GPCR dimer TR-FRET sensor based on anti-HA/flag/c-Myc antibody. **(B)** Conformational changes in TR-FRET sensor of dimeric GPCR using different tags, including N-terminal labeling and the intra-subunit conformational TR-FRET sensor. **(C)** Conformational changes in TR-FRET sensor of GPCRs based on active conformation-binding nanobodies. **(D)** Ribbon model structures of the different tags used to label receptors: IgG (PDB ID: 1IGT), Halo tag (PDB ID: 5UY1), SNAP tag (PDB ID: 3KZZ), and ACP tag (PDB ID: 2MLB), UAA (4-Azido-L-phenylalanine, CAS No.: 33173-53-4). **(E)** Common TR-FRET compatible dyes. Lumi4-Tb was used as donor, and red or green dyes were used as acceptors.

Antibodies labeled with long-lifetime lanthanide-based cryptate fluorophores were used in the first-generation TR-FRET sensors, which target small tags fused in GPCRs, such as HA, Flag, or c-Myc (Figure 6A). It was used to prove the protein interactions and indicate the interface (Kniazeff et al., 2004; Liu et al., 2004). However, it failed to monitor the dynamic changes between the GPCR subunits, might because of the large size of the antibodies.

The SNAP tag is two-thirds the size of GFP, derived from the O^6^-guanine nucleotide alkyltransferase that covalently reacts with benzyl-guanines (BG). The SNAP tag can be specifically and covalently labeled with any fluorophore carried by the BG benzyl group. By using non-permeant BG derivatives compatible with TR-FRET measurements, it allows to identify the dimer of GPCRs (Figure 6B). Using SNAP and CLIP labeling TR-FRET sensors, the mGluRs, which are considered strict homodimers, have been found to be heterodimers between different sub-groups (Doumazane et al., 2011). Meanwhile, SNAP labeling sensors can be used to identify oligomers, such as the GABA_*B*_ receptor, which was previously considered to be heterodimers (Maurel et al., 2008; Comps-Agrar et al., 2011).

Furthermore, a N-terminal SNAP tag strategy combined with TR-FRET technology was developed to monitor the dynamic changes between two subunits in class C GPCR dimer. The mGlu receptors are dimeric entities with a large ECD, and during activation, a large conformational change results from the rotation of almost 70° of one ECD relative to the other (Huang et al., 2011). According to the available structures of mGlu receptor ECDs (Kunishima et al., 2000; Tsuchiya et al., 2002; Muto et al., 2007; Koehl et al., 2019), the distance between the N terminus varies from 2.8 nm in the inactive conformation to 3.3 nm in the active conformation. Consistently, in the TR-FRET measurement, inactive mGlu2 receptors were found to have a high FRET signal, while a low FRET signal was obtained in the active state (Figure 6B). The agonist-induced decrease in inter-subunit FRET efficiency was caused by a large change in the distance between the separated fluorophores (Doumazane et al., 2013). Such a movement is closely related to receptor activation, as agonist potencies presented by TR-FRET are perfectly correlated with those determined in cell-based functional assays (including IP1, cAMP, and Ca^2+^ release), indicating the efficiency and accuracy of the conformational change-sensitive TR-FRET sensor. The N-terminal SNAP strategy is feasible for most class C GPCRs, including all mGlu family members (mGlu1-8) and CaSR (Scholler et al., 2017a; Liu et al., 2020; Figure 6B). SNAP-tagged TR-FRET sensor of the mGlu5 receptor based on ECD conformation showed enhanced basal activation in the presence of D1R (Sebastianutto et al., 2020).

To increase the compatibility of SNAP-tagged TR-FRET sensor to different GPCRs, several modifications are required to obtain a large signal-to-noise ratio, including the insertion position, labeling strategies, and methods to quantify signals. For example, the N-terminal SNAP strategy does not work for the GABA_*B*_ receptor because GABA_*B*_ receptors do not undergo a strong conformational change similar to that of the mGlu receptors during activation (Geng et al., 2012; Lecat-Guillet et al., 2017). To detect the conformational change of this receptor, researchers kept the SNAP tag on the N-terminal of the GABA_*B1*_ subunit and introduced a short version of the acyl carrier protein (ACP)-tag (ACP_17_) within extracellular lobe 2 of the same subunit based on the knowledge of the activation of the GABA_*B*_ receptor (Figure 6B). A high TR-FRET signal was largely decreased upon GABA_*B*_ receptor activation and was suppressed by the competitive antagonist CGP54626 (Lecat-Guillet et al., 2017; Scholler et al., 2017a). Different sizes of tags, such as ACP (8 kDa) and ACP_17_ (2 kDa) (George et al., 2004; Yin et al., 2005) or the O^6^-alkylguanine-DNA alkyl transferase derivatives, SNAP (Keppler et al., 2003), CLIP (23 kDa) (Gautier et al., 2008), and Halo Tag (33 kDa) (Encell et al., 2012), combined with compatible fluorophore pairs build a toolbox for TR-FRET sensors optimization (Figures 6D,E). Another representative application of intra-subunit sensor is the luteinizing hormone (LH) receptor from the class A family, which has a large extracellular domain. After the addition of agonist, TR-FRET signals increased while the ACP_17_ and SNAP tags were constructed at the loop and N terminus, respectively, revealing an important conformational change within the extracellular domain of the LH receptor. The strategy of the extracellular intra-subunit sensor is feasible for these kinds of monomeric GPCRs, in which the extracellular domain undergoes a large conformational change during activation (Scholler et al., 2017a).

In addition to class C GPCR, some classes A and B GPCRs were found to form dimer or oligomer (Carrillo et al., 2003; Berthouze et al., 2005; Harding et al., 2009; Kasai et al., 2018). These heterodimers showed distinct functions and related to pathogenesis. For example, AT_1_R and B_2_R heteromerization was found to occur in human placental biopsies from pregnancies complicated by preeclampsia, and the aberrant heteromerization of AT_1_R-B_2_R was found to result in exaggerated calcium signaling and high vascular smooth muscle mechanosensitivity (Quitterer et al., 2019). TR-FRET sensors are also adaptable to other GPCR dimers, such as class A relaxin family peptide receptor 3 (RXFP3) and the LH receptor, class B PTHR, corticotropin-releasing factor receptor 1 (CRF1R), and pituitary-activating cAMP polypeptide (PACAP) receptor 1 (PAC1). The potency of a series of agonists obtained from measurements of the TR-FRET assay correlated with those obtained in functional assays (Scholler et al., 2017a). On the other side, BRET approaches using Rluc and YFP pair can be used to identify the formations of GPCR dimers (Ayoub and Pfleger, 2010; Johnstone and Pfleger, 2012; El et al., 2019). But few intermolecular BRET conformational change sensors have been reported. Though ligand-induced BRET changes have been presented in MT1/MT2 melatonin receptor heterodimers (Ayoub et al., 2002, 2004), conformational changes in other dimers such as F prostanoid receptor, were not robust enough for investigation (Sleno et al., 2016).

The unnatural amino acid (UAA) site-directed modification strategy is considered to be a potential way to build flexible RET sensors that minimize the labeling tag size into one residue. The UAA technology can be used to investigate the interaction sites between two proteins, such as β-arrestin binding to AT_1_R (Gagnon et al., 2019). Then, with an optimization in UAA incorporation strategy, it can measure FRET signal between two specific labeling sites of GPCR at the living cell level and the single-molecule level (Liauw et al., 2021). This UAA incorporation strategy in living cells provide a good protocol to apply UAA instead of other tags in GPCR sensors, which may have higher sensitivity to monitor more differential conformational change.

## Conclusion and Perspectives

In this review, we summarized four types of conformational sensors for GPCR signaling and activation based on FRET and BRET. These sensors have identified new mechanisms of GPCRs activation process and also lead to significant breakthroughs in high-throughput drug screening toolboxes. Generally, most FRET sensors show strong intensity and microscopy compatibility, which possess better spatial and temporal resolution for imaging purposes. The TR-FRET sensor can also be applied in HTS. BRET sensors have more sustainable signals, higher signal-to-noise ratios and HTS applications. These sensors have been optimized using various labeling strategies to increase the sensitivity and compatibility, from heterogeneous systems to endogenous conditions. Using these assays, GPCR signaling and activation have been investigated on a large scale and at multiple levels. However, the introduction of BRET and FRET sensors without breaking normal expression and function remains challenging. The UAA site-directed modification strategy for FRET sensors may be a promising approach (Liauw et al., 2021). BERKY biosensors for endogenous G proteins will be a good choice to investigate endogenous GPCR activation (Maziarz et al., 2020).

Meanwhile, although smaller and smaller tags can be preferred to minimize the extra influence, a few FRET sensors based on traditional antibodies (Liu et al., 2004; Comps-Agrar et al., 2011) or labeled small molecule ligands (Albizu et al., 2010) have shown advantages in the detection of GPCR in native samples. However, due to the excessive molecular weight of antibodies or insufficient specificity of some antibodies and small molecule ligands, it is difficult to detect GPCR complexes in native tissue. Nanobodies, which have a smaller size, higher affinity, and conformation specificity, may provide breakthroughs in native GPCR functional assays and signaling (Scholler et al., 2017b; Figure 6C). As the hetero-complexes of GPCRs have received increasing attention for their connection with diseases (Prinster et al., 2005; Gomes et al., 2016), nanobody-based assays will provide useful tools for investigating roles of GPCR heteromers in physiological and pathological processes in the future.

## Author Contributions

All authors listed have made a substantial, direct and intellectual contribution to the work, and approved it for publication.

## Conflict of Interest

The authors declare that the research was conducted in the absence of any commercial or financial relationships that could be construed as a potential conflict of interest.

## Abbreviations

β_2_AR: β _2_-Adrenergic receptor
α_2A_ AR: α 2A adrenergic receptor
A_2A_R: A_2A_-adenosine receptor
AT_1_R: angiotensin II receptor type 1A
BRET: bioluminescence resonance energy transfer
FRET: förster resonance energy transfer
GABA_*B*_ receptor: metabotropic γ -aminobutyric acid receptors
GFP: green fluorescent protein
GPCRs: G protein-coupled receptors
HTS: high-throughput screening
ICL: intracellular loop
KOR: κ opioid receptor
LH: luteinizing hormone receptor
mGluR: metabotropic glutamate receptor
P2Y_2_R: Purinergic receptor
PTHR: Parathyroid hormone 1 receptor
Rluc: Renilla Luciferase
TM: Transmembrane helix
TR-FRET: time-resolved FRET
VFT: Venus fly-trap
YFP: yellow fluorescent protein
7TM: seven-transmembrane.
